# Has Uganda experienced any stalled fertility transitions? Reflecting on the last four decades (1973–2011)

**DOI:** 10.1186/s40738-015-0006-1

**Published:** 2015-09-23

**Authors:** Allen Kabagenyi, Alice Reid, Gideon Rutaremwa, Lynn M. Atuyambe, James P. M. Ntozi

**Affiliations:** 1 Department of Population Studies, School of Statistics and Planning, College of Business and Management Sciences, Makerere University, P.O. Box 7062, Kampala, Uganda; 2 Center for Population and Applied Statistics, College of Business and Management Sciences, Makerere University, P.O. Box 7062, Kampala, Kampala Uganda; 3 Department of Geography, University of Cambridge, Downing Place, Cambridge, CB2 3EN UK; 4 United Nations Economic Commission for Africa, Social Development Policy Division, P.O. Box 3001, Addis Ababa, Ethiopia; 5 Department of Community and Behavioral Sciences, School of Public Health, College of Health Sciences, Makerere University, P.O. Box 7062, Kampala, Kampala Uganda

**Keywords:** Stalled fertility, Transition, Socioeconomic factors, Demographic factors, Uganda

## Abstract

**Background:**

Persistent high fertility is associated with mother and child mortality. While most regions in the world have experienced declines in fertility rates, there are conflicting views as to whether Uganda has entered a period of fertility transition. There are limited data available that explicitly detail the fertility trends and patterns in Uganda over the last four decades, from 1973 to 2011. Total fertility rate (TFR) is number of live births that a woman would have throughout her reproductive years if she were subject to the prevailing age specific fertility patterns. The current TFR for Uganda stands at 6.2 children born per woman, which is one of the highest in the region. This study therefore sought to examine whether there has been a fertility stall in Uganda using all existing Demographic Health Survey data, to provide estimates for the current fertility levels and trends in Uganda, and finally to examine the demographic and socioeconomic factors responsible for fertility levels in Uganda.

This is a secondary analysis of data from five consecutive Ugandan Demographic Health Surveys (UDHS); 1988/1989, 1995, 2000/2001, 2006 and 2011. Using pooled data to estimate for fertility levels, patterns and trends, we applied a recently developed fertility estimation approach. A Poisson regression model was also used to analyze fertility differentials over the study period.

**Results:**

Over the studied period, fertility trends and levels fluctuated from highs of 8.8 to lows of 5.7, with no specific lag over the study period. These findings suggest Uganda is at the pre-transitional stage, with indications of imminent fertility rate reductions in forthcoming years. Marital status remained a strong predictor for number of children born, even after controlling for other variables.

**Conclusions:**

This study suggests there is no evidence of a fertility stall in Uganda, but demonstrates an onset of fertility transition in the country. If this trend continues, Uganda will experience a low fertility rate in the future—a finding pertinent for policy makers, especially as the continent and the country focus on harnessing the demographic dividend.

## Background

Persistent high fertility remains a public health concern as it impedes efforts to reduce maternal and child mortality [1–3]. Fertility decline, particularly in sub-Saharan Africa, has been debated among scholars, highlighting divergent views pertaining to fertility transition [4, 5]. Most regions of the world have experienced gradual fertility declines, the latest starting in the 1940s. Compared with other regions such as Asia and Latin America, sub-Saharan Africa lags behind these trends, with a Total Fertility Rate (TFR) of 5.1 children born per woman [6]. This rate is far above the other regions that have already reached replacement fertility [7, 8]. The United Nations have estimated a drastic reduction fertility levels or population growth in these regions since the onset of fertility transition in the early 1960s [9]. Variation and pace of fertility decline in different regions and countries has been identified [11, 12], and to some researchers sub-Saharan Africa is the last region in the world to begin fertility transition [10].

Bongaarts [13] using multiple survey data made an explicit illustration of the transition and identified seven countries (Bangladesh, Turkey, Dominican Republic, Colombia, Peru, Kenya and Ghana) with stalled fertility; two of these were countries in sub-Saharan Africa. The Bongaarts study defined stalled fertility as countries with no significant reduction between recent or consecutive surveys. The fertility stall in the mid-1990s ranged from 4.7 births per woman in Kenya to 2.7 births per woman in Turkey. The other countries (Uganda, Mali, Burkina Faso, Mozambique, and Niger) were not considered because they were above the pre-transitional stage, implying the latter countries had not yet begun their visible fertility transition. These countries belong to a region of Africa that has a persistently high, yet unmet need for contraception, as well as preference for a large family size [12, 14, 15]. Additionally, high fertility rates have also been attributed to low use of contraception [16, 17] and sociocultural inhibitions [18, 19].

Divergent views have been raised explaining stalling fertility as a new type of transition [20], owing to changes in older women’s fertility and a longer postpartum period [21]. Bongaarts [17] argued that more than half of all sub-Saharan countries had stalled fertility. Additionally, Garenne [4] and Ezeh et al. [5] highlighted a stall in fertility in Eastern African countries associated with increased adolescent fertility and larger family size preference. Schoumaker [22] noted that the fertility stall in sub-Saharan Africa was spurious compared with other countries with more consistent declines in fertility. There have been successes identified in a few African countries, specifically Rwanda, Malawi, Madagascar and Ethiopia, which have had a consistent decrease in fertility and unmet need for contraception [23, 24]. Unmet need is defined as the gap between expressed fertility preference and actual contraceptive use [44]. The exceptionally robust family planning programs and continued awareness and availability of contraception have been identified as possible reasons for the consistent decrease in fertility [16, 26]. Some researchers suggested the stalls are superficial, leading to a risk of reversal in the fertility rate reductions, as observed with Kenya’s fertility transition [27].

It is still unclear as to why the fertility rate seems to have stalled in some African countries—even after having substantial fertility declines—while in others the birth rates continue to be high. Most previous studies have examined fertility stall using customized Demographic and Health Survey (DHS) tabulations, where multiple countries at a time are examined using two to three surveys [5, 13, 16, 28]. There is lack of country-specific information or studies that explicitly show the direction of fertility over retrospective years. Few examples exist of country-specific analysis [29–32], and these studies have analyzed cross-sectional surveys; therefore, the fertility stall could not be fully examined. In comparison, the present study seeks to explore fertility rates over a 40-year period. The robust methodology adopted in this study is a technique suggested by Schoumaker [33], which has not been used before for Uganda and other high fertility countries. A similar technique, suggested by Garenne [34] has proven to be successful in examining longer periods of fertility patterns and trends and explicitly determines whether there is a fertility stall.

Bongaarts [13], while examining the causes of stalling fertility, used DHS tabulations to explore fertility reductions for a number of sub-Saharan countries. While similar studies have examined fertility transition in sub-Saharan Africa while examining multiple countries, the current study analyzes the levels, trends and patterns of Uganda’s fertility since 1973, to determine whether the country has experienced stalling fertility. This study further explores to what level the fertility transition stage has reached and the demographic and socioeconomic factors determining the pace of transition. As all countries and regions are unique, the method used maintains the integrity of country-specific data, avoiding combining cross-country data while assessing these demographic indicators [30–32]. There is currently no information showing country-specific analysis that could explicitly explain the fertility stall or reduction in Uganda. This study therefore sought to: i) examine whether there is a fertility stall in Uganda using all existing DHS data; ii) provide estimates for the current fertility levels and trends in Uganda; and iii) examine the demographic and socioeconomic factors associated with fertility levels in Uganda.

## Methods

This study is based on the technique of reverse survival using information from the children of each mother. This technique provides an estimation of annual age-specific fertility rates for a period of 10 to 15 years before any survey or census. The analysis was based on information obtained from the number of children classified by the age of the mother at the time of the survey. We used the Schoumaker [33] method of retrospective fertility estimation, which was deemed appropriate to provide robust patterns, trends and levels of TFR in any given geographical area. In the latter approach the children are linked to their mothers using reverse projection to the time of their births, classified by the age of the mother. This approach has been used elsewhere successfully to estimate fertility [30, 34]. The method can also be used to estimate differentials in fertility, using rate ratios.

### Data source

Data were sourced from all existing Uganda Demographic and Health Surveys (UDHS) conducted since 1988. The cross-sectional surveys are part of the worldwide DHS project that collects data on comparable demographic and health indicators in selected developing countries. The sample sizes of the surveyed women, aged 15–49 years in Uganda were; 4857 in 1988/89; 7070 in 1995; 7246 in 2000/01; 8531 for 2006 and 8674 in 2011 [38–43]. These samples were obtained using a two-stage cluster sampling process. This began with the selection of clusters or enumeration areas from a list of clusters generated by the Uganda Bureau of Statistical (UBOS) followed by the selection of households from each cluster. The data collected were also stratified by rural and urban areas. Details of the response rates for the eligible and interviewed women are presented in Table 1.

**Table 1:** Summary of the sampling and response rate for the conducted DHS

Year of survey	Enumeration Area	Number	(%) Response rate	
			Household	Women
2011	405	8674	95.3	93.8
2006	404	8531	97.5	94.7
2000/2001	298	7246	95.8	93.9
1995	295	7070	98.4	95.8
1988/1989	206	4857	91.3	97.4

Note: DHS is demographic and health survey

DHS data were used because they include birth histories of the mother, where the dates of births of all children born to a mother are reported. However, this method can be limited by adoption and misreporting of age, although it was considered to provide the best available data, when accurate age was reported.

### Data included

Information on children under the age of 10 or 15 years before the survey, classified by single year ages. Specifically:

1. Date of birth for each respective child;
2. Date of birth for each woman, irrespective of whether the woman had ever given birth and
3. The date of the survey.

### Estimated output parameters

- The respective age-specific fertility rates for the three years preceding the survey and
- Total fertility for each of the 10 or 15 years preceding the survey.

### Analysis and reconstruction of fertility trend

Using five DHS data sets for Uganda for the period 1988/89 to 2011, data quality was assessed for age and date of birth for the children and their respective mothers. This is important to control for age heaping and misreporting which has been reported in demographic and health surveys data [22].

Country-specific demographic indicators were provided and estimate for age-specific fertility rates and TFRs for women for three-year period prior to the survey. Subsequently, fertility reconstruction was performed for over 38 years, using a method proposed by Schoumaker [33], based on pooled Ugandan DHS data. This method employed the person-period approach to analyze all the birth histories of the women, using a Poisson regression model:

$$ \mathrm{Log}\ \left({\upmu}_{\mathrm{i}}\right) = \log\ \left({\mathrm{t}}_{\mathrm{i}}\right) + \upalpha +f\left(\mathrm{age}\right) + \mathrm{g}\left(\mathrm{time}\right), $$

where:

μ_i_ is the expected number of children born to the mother in each respective time segment;

t_i_ is the length of the time or exposure;

*f* (age) is a function of estimated age; and

*g* (time) is a function of calendar time.

Age is a dummy that represents the 5-year age groups of the mother, and calendar time represents annual fertility variations for the respective Ugandan DHSs. The yearly total fertility estimates are presented accordingly in the Table 2. Linear regression was used to identify associations in the data. The simple linear regression takes the form of:

**Table 2:** Retrospective total fertility rates (TFRs) by single calendar years for the period 1973–2010 at 95 % confidence interval

					DHS year					
Years	1988/89		1995		2000/01		2006		2000/01	
1973	7.72	(7.00-8.43)								
1974	8.30	(7.59-9.02)								
1975	7.61	(6.95-8.26)								
1976	8.78	(8.10-9.46)								
1977	7.18	(6.58-7.77)								
1978	7.98	(7.37-8.58)								
1979	7.65	(7.08-8.22)								
1980	8.36	(7.78-7.78)	8.53	(7.93-9.13)						
1981	6.80	(6.29-7.30)	6.66	(6.14-7.16)						
1982	8.09	(7.55-8.63)	8.05	(7.51-8.59)						
1983	7.25	(6.76-7.75)	7.37	(6.87-7.87)						
1984	7.03	(6.55-7.50)	7.07	(6.60-7.54)						
1985	7.30	(6.83-7.77)	7.78	(7.31-8.26)	7.19	(6.64-7.74)				
1986	7.32	(6.86-7.78)	7.72	(7.26-8.18)	8.19	(7.62-8.75)				
1987	7.35	(6.90-7.80)	7.47	(7.03-7.90)	7.54	(7.02-8.06)				
1988			7.36	(6.94-7.78)	7.79	(7.28-8.30)				
1989			7.18	(6.78-7.59)	6.98	(6.52-7.45)				
1990			8.57	(8.14-9.01)	7.71	(7.23-8.19)				
1991			5.93	(5.58-6.27)	6.79	(6.37-7.22)	7.21	(6.73-7.69)		
1992			6.80	(6.44-7.16)	7.93	(7.47-8.38)	8.36	(7.86-8.86)		
1993			7.01	(6.65-7.37)	7.54	(7.11-7.97)	7.70	(7.24-8.17)		
1994			7.73	(7.36-8.10)	8.61	(8.16-9.07)	8.53	(8.06-9.00)		
1995					6.39	(6.01-6.76)	7.47	(7.04-7.90)		
1996					7.36	(6.96-7.75)	8.15	(7.72-8.59)	8.22	(7.70-8.73)
1997					7.23	(6.86-7.61)	7.22	(6.83-7.62)	7.54	(7.07-8.02)
1998					6.66	(6.31-7.01)	7.72	(7.32-8.12)	7.89	(7.42-8.36)
1999					7.30	(6.94-7.67)	7.68	(7.29-8.07)	7.72	(7.27-8.17)
2000							8.47	(8.07-8.87)	8.25	(7.80-8.70)
2001							6.30	(5.97-6.64)	6.86	(6.47-7.26)
2002							7.24	(6.89-7.60)	6.97	(6.58-7.36)
2003							6.92	(6.58-7.26)	7.87	(7.47-8.28)
2004							6.66	(6.3-6.98)	7.31	(6.93-7.69)
2005							6.69	(6.37-7.01)	7.58	(7.20-7.96)
2006									6.76	(6.41-7.10)
2007									6.52	(6.19-6.85)
2008									6.72	(6.39-7.05)
2009									6.43	(6.11-6.74)
2010									6.06	(5.76-6.36)
2011										

Note: UDHS is Uganda Demographic and health Survey

$$ {\gamma}_i=\alpha + {\beta}_{\chi_i}+{\varepsilon}_i $$

γ_i =_ this is the value of the dependent variable in observation i, α is a constant, x_i_ is the independent or the explanatory variable in observation i. The coefficient $\beta$ (also known as the slope) measures the gradient of the regression line.

To identify factors associated with fertility decline, rate ratios for the socioeconomic indicators were calculated for the two UDHSs (1988/89 and 2011) weighted data. Rate ratios were calculated with an assumption of proportionality of rates, that the age pattern of fertility is similar or constant across the different categories. Categorical variables are used as covariates in the model and age-specific fertility rates were calculated for the respective reference categories and rate ratios of the other categories and variables. These calculations were performed using the adopted tfr2 approach for all the surveys using categorical variables among all women. The rate ratios can be interpreted as ratios of TFRs [33].

Ethical approval was granted by the Uganda National Council of Science and Technology (UNCST), and the School of Statistics and Planning Higher Degrees Ethical Research Committee. Permission was also sought from Measure DHS to access all the data required.

## Results

Estimates of retrospective TFR and selected socioeconomic and demographic indicators in Uganda are presented in Table 3. The country’s TFR has remained persistently high, above 6, particularly among rural women whose rate often exceeds 7 children per woman. Urban women’s TFR levels have gradually been reducing from 5.7 in 1988/89 to 3.8 in 2011. All methods of contraception have increased 4.9 % in the 1988/89 surveys to 30 % in the 2011 survey. The increase has been most evident among users of modern contraception including; condoms, pills, injectables, implants, intra uterine device (IUD), female sterilization, foam and diaphragm. Age at first marriage has remained particularly low at 17 years, which exposes women to a longer duration of childbearing years. Age at first sexual intercourse of women increased slightly from 15.6 years in 1988 to a current estimate of 16.8 as of the 2011 survey.

**Table 3:** Selected socioeconomic indicators for Uganda for the period 1988–2011

Characteristics	Demographic and health survey				
	1988-89	1995	2000-01	2006	2011
TFR	7.4	6.9	6.9	6.7	6.2
Residence					
Rural	7.6	7.2	7.4	7.1	6.8
Urban	5.7	5.0	4.0	4.4	3.8
Contraceptive use					
Any Method	4.9	14.8	22.8	23.7	30
Modern method	2.5	7.8	18.2	17.9	26
Median Age at First sexual Intercourse	15.6	16.0	16.6	16.4	16.8
Median age at first marriage	17.0	17.4	17.8	17.6	17.9

### TFR and age-specific fertility rates for three years preceding the DHS

**Fig. 1:**
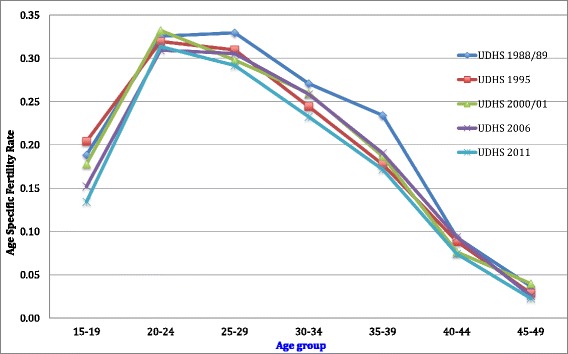
Age-specific fertility levels for the 3-year period preceding each survey (1989–2011)

**Fig. 2:**
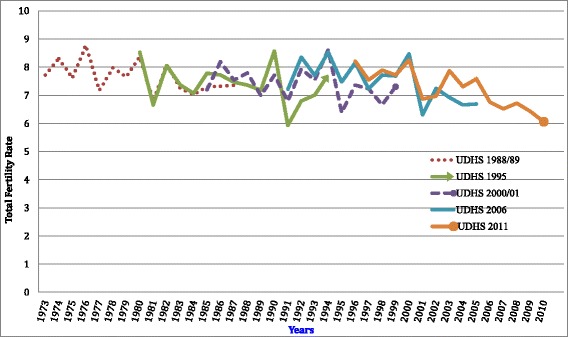
Retrospective fertility rates using the own children method for a single calendar years using consecutive UDHS (1988/89–2011)

Age-specific fertility levels for the three year period preceding the DHS are presented in Fig. 1. Rates reduced in each age group, with the highest rates being recorded in the 1988/89 survey compared with the 2011 survey. TFR has been consistent for the corresponding DHSs, as indicated in Fig. 2.

### Retrospective fertility levels

Retrospective annual TFR estimates for a 15-year period, for each survey are presented in Table 2. The same trends over the years are illustrated in Fig. 2, providing visible assessment of the annual fertility rates for a period of 38 years.

**Fig. 3:**
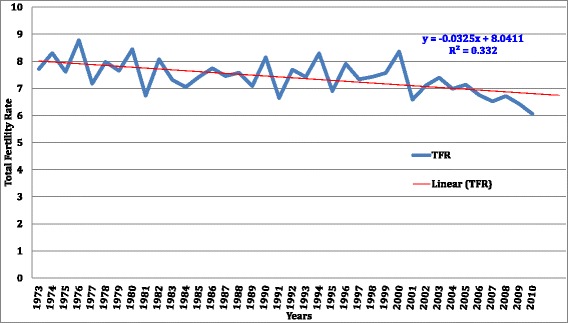
Reconstructed fertility trends of women aged (15–49) of Uganda for 37 years based on DHS surveys

The regression analysis shows variations in the fertility rates, ranging from a high of 8.8 in 1976 to a low of 6.2 in 2010 (Table 2). For the 15-year period prior to the first DHS of 1988/89, TFR was 7.7 children per woman born in 1973, increasing to 8.3 in 1974, and reducing to 7.6 in 1975. There was a reduction from a TFR of 8.4 in 1980 to 6.8 in 1981. The TFR remained as high as 7 until 1987. Overall, during the period before 1995, TFR varied between 8 and 7, although in 1991 an estimate of 5.9 was observed. For the most recent survey in 2011, TFR estimates ranged from approximately 8 in 1996 to 6.1 in 2010. It is only during this latter period that a relatively consistent TFR of 6 was observed without major fluctuations. Owing to the observed TFR variability over the study period a linear equation was fitted to the average estimates as presented in Fig. 3 and analyzed in the subsequent sections of this manuscript.

### Fertility trends over the 38 year period

Average fertility estimates for each year are presented in Fig. 3. These results are presented with the fitted regression line to show the TFR trend over time. Overall, the TFR in Uganda has been high, ranging from 8 in the 1970s to 6 in 2010. The regression line shows a gradual reduction for the study period, with a possibility of continued reduction in subsequent years. The results also show no fertility stall in the close four-decade period examined.

### Fertility differentials for 1988/89 to 2011

The results for the fertility differentials in Uganda for the 3 years preceding the survey are presented in Table 4. The results display TFR and age specific fertility rates of the reference category which is no education while the rate ratios are presented for all the other categories and covariates. The results show the net effect of education on fertility, controlling for place of residence, marital status, wealth status and contraceptive use. Marital status remained consistent with a strong influence on total fertility compared with all the other variables. The fertility rate has been increasing in married women, as evidenced by the increasing rate ratios from 3.8 in 1988/89, 4.6 in 1995, 5.9 in 2001, 6.4 in 2006 and 6.7 in 2011. Additionally, the results show that women with secondary and primary education had an influence on fertility levels.

**Table 4:** Socioeconomic and demographic fertility differentials for 1988/89 to 2011 based on DHS surveys

Variable	DHS Year														
	1988/89			1995			2000/01			2006			2011		
Age	Coef.	95 % CI		Coef.	95 % CI		Coef.	95 % CI		Coef.	95 % CI		Coef.	95 % CI	
15-19	0.07	0.05	0.08	0.05	0.04	0.06	0.04	0.03	0.05	0.05	0.04	0.05	0.04	0.03	0.04
20-24	0.09	0.07	0.11	0.06	0.05	0.07	0.05	0.04	0.06	0.06	0.05	0.07	0.05	0.04	0.07
25-29	0.09	0.07	0.11	0.06	0.05	0.07	0.04	0.03	0.05	0.05	0.04	0.06	0.04	0.04	0.05
30-34	0.07	0.05	0.09	0.04	0.04	0.05	0.04	0.03	0.04	0.04	0.03	0.05	0.04	0.03	0.04
35-39	0.06	0.05	0.08	0.03	0.03	0.04	0.03	0.02	0.03	0.03	0.02	0.04	0.03	0.02	0.03
40-44	0.02	0.02	0.03	0.02	0.01	0.02	0.01	0.01	0.01	0.02	0.01	0.02	0.01	0.01	0.01
45-49	0.01	0.01	0.01	0.01	0.00	0.01	0.01	0.00	0.01	0.00	0.00	0.01	0.00	0.00	0.01
	Rate ratios														
	DHS year														
Variable	1988/89			1995			2000/01			2006			2011		
Education															
None^Rc^	1.0			1.0			1.0			1.0			1.0		
Primary	0.97			1.07**			1.01			1.05			1.06		
Secondary	0.93			.95			.78***			0.96			1.04		
Residence															
Urban^Rc^	1.0			1.0			1.0			1.0			1.0		
Rural	1.11*			1.23***			1.3***			1.12**			1.17**		
Marital Status															
Single^Rc^	1.0			1.0			1.0			1.0			1.0		
Married	3.8***			4.6***			5.9***			6.4***			6.71***		
Formerly	2.8***			3.1***			3.6***			4.2***			4.55***		
Wealth Index															
Poorest^Rc^							1.0			1.0			1.0		
Poorer							0.98			0.96			0.91**		
Middle							1.10**			0.94			0.92*		
Richer							1.08*			0.93			0.88***		
Richest							1.05			0.75***			0.71***		
Contraceptive use															
Not Using ^Rc^	1.0			1.0			1.0			1.0			1.0		
Using method	1.12			1.11**			1.06*			0.93*			1.00		

* *p* < 0.1; ** *p* < 0.05; *** *p* < 0.01 , ^Rc^ Reference Category

The net effect of education on these variables is evident in the 1995 and 2000/01 survey periods, where in 1995, women with primary education had rate of 1.07 compared with those with no education. Those with secondary education had a reduced rate ratio of 0.78 in 2000/01. Even after controlling for other variables, place of residence had a significant effect on fertility levels, particularly among rural women who had higher fertility rates compared with those residing in urban areas. These were presented as rates ratios of 1.1 in 1988/89, 1.2 in 1995, 1.3 in 2000/01, 1.1 in 2006 and 1.2 in 2011.

Information on the variable wealth status was not collected in the two retrospective surveys of 1988/89 and 1995 therefore these data are missing in the output. Wealth status had a strong effect on fertility, particularly in the most recent survey of 2011, while contraceptive use was an important influence in the 1995, 2000/01 and 2006 surveys. The rates for contraceptive use compared with those who were not using any method were 1.1 (1995), 1.06 (2000/01) and a reduced effect of 0.93 in 2006.

## Discussion

This paper aimed to analyze the rates, trends and patterns of Uganda’s fertility since 1973 and to determine whether the country’s fertility rate has stalled. This manuscript explored the stage of fertility transition Uganda has reached as well as the demographic and socioeconomic factors determining the pace of fertility transition. In doing this we used all the existing DHS data on the country. Our key finding was that for the last four decades there has been no indication of a fertility stall; fertility rates declined for a period of time then remained constant. For the study period, TFR did not depict a steady pattern but was fluctuating between highs and lows, with no specific time lag duration. The latter conforms to the divergent views and findings regarding fertility stall, particularly among sub-Saharan countries [5, 16, 17, 35].

This study highlights that the country is at a pre-transitional stage, with indications of imminent reductions in TFR in subsequent years. Our findings are supported by the work of Bongaarts [13, 17], who indicated the country was still in the pre-transitional stage. However, in divergence with the reports of fertility stall as reported by Bongaarts [13] and Ezeh [5]. The high TFR in Uganda could be explained by the young age at marriage, which has remained considerably low in the country [40–42]. Additionally, the study highlights there has been limited variability in the tempo of fertility for the study period, as depicted by the Age Specific Fertility Rate (ASFR) patterns. Whereas the tempo effect could be influenced by sampling variability, it could also be a reflection of the true fertility outcomes in the population. There also a clear indication the country still experiences a high birth phenomenon, which is important in understanding the fertility transition in Uganda. Previous research in Bangladesh identified tempo and fertility have a significant effect on the levels and trends in the population fertility transition [32].

The findings of this study have important policy implications, especially in addressing these issues in a young and growing population. The country should embrace the opportunity to invest in contraceptive programs to accelerate fertility reduction. The increased investment in family planning and its effect on fertility declines have been well documented elsewhere by Westoff and Cross [31], in addition to the positive effects of investment in female education [10] and socioeconomic development Bongaarts [13]. It is possible that stalling fertility exists in rural or urban areas or there are regional differences as suggested by Ezeh [5]; however, these have not been explored in our study. Therefore we propose the contribution of rural and urban fertility transition, and the potential influence on fertility rates of the country is explored in further studies.

The socioeconomic and demographic factors examined, including marital status, education, residence and wealth status had a significant influence on the levels of fertility. For instance, marital status remained a strong predictor, even after controlling for other variables, which explains the role of marriage on the number of children born. Women give birth to more children within a marriage compared with when they are single [18, 21, 36, 37]. The influence of marital status on fertility outcomes could be explained in relation to age at marriage. Marrying at a younger age exposes women to the associated risks of having more children, in addition to the low use of contraception in Uganda. Modern contraceptive use did not have an influence on fertility rate, potentially because uptake is too low to make significant differences in the general population [25, 31].

Additionally, women living in rural areas had increased fertility rates compared with those living urban areas. The influence of residence cannot be underestimated, given the limited access to adequate health care services, information, family planning messages and education in these areas [20, 23].

There is a need for deliberate government efforts to encourage women to delay marriage and increase uptake of modern contraception in Uganda. Education level, particularly for women, should be enhanced further—up to at least secondary education. Without political involvement to reinforce these key demographic indicators, the situation will not change and high fertility levels will take considerable time to reduce to manageable numbers.

The strength of the analysis in this manuscript is that it is based on nationally representative demographic data. The rigorous measures used in the analysis were developed recently for birth history measurement and analysis [33]. Further the method used facilitates computation of fertility rates, evaluates data quality, reconstruction of annual fertility trends and estimation of fertility levels and trends [33]. The measurement however, is limited to only the aforementioned analyses and not be used to estimate for parity specific fertility rates and parity progression ratios.

## Conclusions

The findings suggest there is no fertility stall in Uganda but demonstrates an onset of fertility transition where the levels are likely to continue to decline consistently. This study is relevant for policy makers, particularly at this point in time when the country is focusing on embracing the demographic dividend. This occurs when a country has accelerated economic growth resulting from rapid decline of the fertility rates, which affects population age structure. As a reduction in fertility commences, the country ought to facilitate this process with increased investment in education and family planning. This study is the first to have reconstructed fertility levels and trends over a 40-year period, and has established Uganda is commencing a period of fertility transition.

## Abbreviations

ASFR: Age specific fertility rates
DHS: Demographic and health survey
IUD: Intra uterine device
TFR: Total fertility rate
UDHS: Uganda demographic and health survey
UNCST: Uganda national council of science and technology
